# Perception of Preparedness of Health Care Professionals in Case of a Nuclear, Chemical, Biological Attack/Emergency in a Tertiary Care Hospital

**DOI:** 10.7759/cureus.4657

**Published:** 2019-05-14

**Authors:** Abdul R Azeem, Muhammad Waqar Sharif, Ali Akhtar, Chaudhry Saad Sohail, Ahmed A Dar, Maham Khan, Maira Aleem

**Affiliations:** 1 General Medicine, Combined Military Hospital, Lahore, PAK; 2 Internal Medicine, Combined Military Hospital - CMH - Lahore Medical College and Institute of Dentistry, Lahore, PAK; 3 Internal Medicine, Pakistan Air Force Hospital, Islamabad, PAK; 4 Internal Medicine, Banner University Medical Center, Tucson, USA; 5 Dentistry, De'montmorency College of Dentistry/ Punjab Dental Hospital, Lahore, PAK; 6 Radiology, Armed Forces Institute of Radiology and Imaging, Islamabad, PAK; 7 Biotechnology, Combined Military Hospital - CMH - Lahore Medical College and Institute of Dentistry, Lahore, PAK

**Keywords:** attack/incident, biological, chemical, health care, nuclear, professional, perception, preparedness

## Abstract

Background

With the growth of global terrorism and rapid advancements in the field of science, the threat of a nuclear, biological, and chemical (NBC) attack remains imminent. This study assesses perceptions of preparedness of health care professionals in case of an NBC attack/incident in a tertiary care hospital.

Patients and methods

We conducted a descriptive, cross-sectional study of 200 health care workers (including nurses and doctors) in a tertiary care hospital, from October 2018 through December 2018. Participants answered 17 yes/no questions and five 5-point Likert scale questions. We analyzed the data using chi-square tests and one-way analysis of variance.

Results

Most participants (73.6%) reported availability to an isolation facility, and a majority of participants (72%) reported they had access to ventilators. Approximately 60% of participants reported they had access to beds, and 44.6% reported access to a laundry facility. Most participants (65.3%) knew of an employee assistance program while 31.1% did not know about such a program at their institution. More than 50% of the respondents think they can deal with an emergency involving an NBC attack while 60% of the respondents did not think that their institution would be able to protect them in the event of an NBC attack/incident.

Overall, the participants were not adequately prepared for a mass scale NBC incident. The level of preparation was linked to the number of courses and training programs completed by the participants, with postgraduate medical personnel having the maximum level of preparedness, followed by medical graduates and nursing personnel.

Conclusion

Given the inadequate level of preparedness for an NBC incident as indicated by our findings, drills and seminars on large-scale emergencies such as an NBC attack should be included in the curriculum of undergraduate medical and nursing students in order to impart them the necessary training and confidence in dealing with an NBC incident.

## Introduction

The nuclear explosions in Hiroshima and Nagasaki illustrated the massive destructive potential of nuclear weapons [1]. Similarly, the devastating effects of nuclear accidents in Chernobyl and Fukushima left the world in awe of the dangers of nuclear power. South Asia alone has an estimated 650 nuclear warheads [2]. As Pakistan is situated in one of the most heavily armed nuclear zones in the world, health care professionals, especially those in military hospitals, must be well aware and well equipped to deal with casualties and victims of nuclear, biological, and chemical (NBC) attacks and provide necessary care to patients while concurrently adopting the required safety measures for themselves.

Recently, terrorist attacks using biological agents like anthrax spores have made bio-terrorism a leading concern at the global landscape. These attacks emphasize the need to detect and eliminate threats in the shortest possible time [3]. Furthermore, advances in biotechnology and microbiology have enhanced the threat of a biological attack [4]. A high-grade biological agent dispersed in a public gathering or a building full of people can cause widespread fatalities. One report suggests that, in increasing weather conditions, anthrax has the potential to be 20% more lethal than a 12.5-kiloton nuclear bomb [5], while another report suggests that 110 kg of anthrax can produce effects equivalent to a 1-megaton hydrogen bomb if used in a densely populated city [6]. For preparedness against these agents, physicians need complete knowledge of the disease and training facilities to protect themselves and provide care for their patients.

Chemical warfare is one of the more lethal human-made threats [7]. In 1997, a chemical weapons convention was signed to control the production and procurement of chemical weapons [2]. Despite this precaution, chemical weapons were used excessively in the Syrian conflict. Given that many of these weaponized chemicals have industrial purposes, the prohibition of the production of many of these compounds is impossible [8]. Some of these agents can be as devastating as a nuclear bomb and cause long-term morbidity and terrible psychological effects [9]. The characteristics of various chemical agents, their toxic effects on the human body, and their decontamination and treatment methods are important for a health care professional to know to protect herself and her patients in an hour of need [10].

Given recent terror incidents in different parts of the world and ongoing war against terrorism, it is vital that we do not underestimate the threat of Nuclear Chemical Biological incident in the South-East Asian subcontinent. The preparedness levels of health care professionals in the hour of need and their confidence to deal with mass scale casualties and injuries is crucial for averting further injury and death. Similarly, the psychological fallout from these incidents can cause lasting trauma, especially for first responders and health care professionals. Therefore, we conducted this study to evaluate the levels of disaster preparedness and resource awareness among our health care professionals.

## Materials and methods

We conducted a descriptive cross-sectional study on 200 health care workers with a 95% confidence interval and a 5% margin of error. Study participants were randomly selected through stratified sampling technique from a field of nearly 1,000 potential candidates. Our sample consisted of three strata: medical graduates, postgraduates, and nurses.

The study was conducted using 200 comprehensive questionnaires distributed to eligible respondents to ensure an adequate percentage response rate. Verbal informed consent was obtained from all respondents, who were informed of the objectives of the study and ensured anonymity (i.e., only group-level findings are reported). The study was conducted at a tertiary care hospital from October 2018 through December 2018.

Ethical review permission was granted by the ethical review board of Combined Military Hospital (CMH) Lahore Medical College. We used a modified version of the questionnaire used in a similar study conducted in Canada after acquiring permission from the corresponding author of the Canadian study [11]. The questionnaire consisted of questions on the perceived institutional adequacy of supplies and stores in event of an NBC attack or accident, routine supports at the institution, support by the institution in case of an NBC attack, and the participants’ confidence in dealing with an NBC attack. The respondents were asked to rate their feelings of professional preparedness in case of an NBC attack or accident, their confidence after the 2010 earthquake, and their perceived institutional preparedness in case of a mass NBC attack.

The questionnaire had 22 questions; 17 had a Yes or No answer structure, the other five questions required selecting from a 5-point Likert scale (1 = Not at all, 2 = Rarely, 3 = Neutral, 4 = Often, 5 = Very much). All data were analyzed in IBM SPSS Statistics for Windows, Version 21.0 (IBM Corp, Armonk, NY).

Quantitative variables were measured by descriptive statistical analysis. Their means and standard deviations were measured, and results were described as percentages. One-way analysis of variance was used to determine statistical significance between the variables. Total scoring of the Likert scale variables was followed by post hoc tests to see significant statistical differences. Finally, we made a means plot to check the homogeneity of our results.

## Results

Of 193 participants (response rate, 96.5%), 99 (51.3%) were men and 94 (48.7%) were women. There were 85 nurses with a Bachelor’s degree in nursing (BSN), 57 participants with Bachelor of Medicine, Bachelor of Surgery (MBBS) degrees, and 51 participants (26.4%) with Fellow of College of Physicians and Surgeons Pakistan (FCPS) degrees.

Most respondents indicated an awareness of accessible resources; 97.4%, 97.4%, 95.3%, and 88.1% of respondents reported awareness of the accessibility of gloves, gauzes, masks, and gowns, respectively. Similarly, 73.6% were aware of available isolation facilities, 72% were aware of available ventilators, 60.1% were aware of available beds, and 44.6% of respondents were aware of available laundry facilities. Most participants expressed confidence in the adequacy of supplies and resources at their institution.

The perceived institutional adequacy of routine supports was rated lower than the routine supplies. We found that 74.1% the health care professionals thought emergency food and water would be provided by the institution in the event of an NBC attack, while 23.3% thought food and water would not be provided by the institution, and 2.6% were unsure. Over half of our respondents (65.3%) knew about an employee assistance program while 31.1% did not know about such a program at their institution. More than 80% of the respondents indicated their facilities did not have internet connectivity. Almost 80% of the respondents reported access to grief counseling. Table 1 presents the response scores for respondents with BSN, MBBS, and FCPS.

**Table 1:** Self-reported responses to potential NCB incidents/attack by education level. Responses were recorded via 5-point Likert scale. All p-values for the above data were found to be 0.00. BSN: Bachelor’s Degree in Nursing; MBBS: Bachelor of Medicine, Bachelor of Surgery; FCPS: Fellow of College of Physicians and Surgeons Pakistan; SD: Standard Deviation; NCB: Nuclear, Chemical, and Biological.

Statement	BSN Mean (SD)	MBBS Mean (SD)	FCPS Mean (SD)
Do you feel adequately equipped to work during chemical attack/incident?	3.34 (1.04)	4.08 (1.3)	4.07 (1.03)
Are you adequately trained to deal with nuclear attack/incident?	3.21 (1.0)	3.89 (1.53)	4.03 (1.11)
Does your institution have adequate programs and policies to respond to a large-scale emergency?	3.03 (1.11)	4.07 (1.44)	3.52 (.67)
In general, since the 2005 earthquake, do you feel confident that our health care institutions are prepared for future earthquakes?	2.91 (1.04)	3.45 (1.11)	3.90 (50)
As a health care professional, do you feel confident that our health care system will protect you during a large-scale infectious disease outbreak or any kind of natural or human-made disasters?	3.10 (1.03)	2.15 (67)	2.37 (82)

In terms of global monitoring of infectious diseases, 52% of the participants reported their institutions lacked any means to update staff on the global status of infectious diseases. Most respondents (76%) had received education on emergency management and planning, while 20% had not received such information. Given our sample location is a general hospital with regular disaster management drills, this response was not surprising. More than 50% of the respondents indicated their institution lacks the means for child care and elder care support in the event of an NBC incident. More than 90% of the participants reported their institutions had no pet care support programs in case of a mass casualty event.

**Figure 1:**
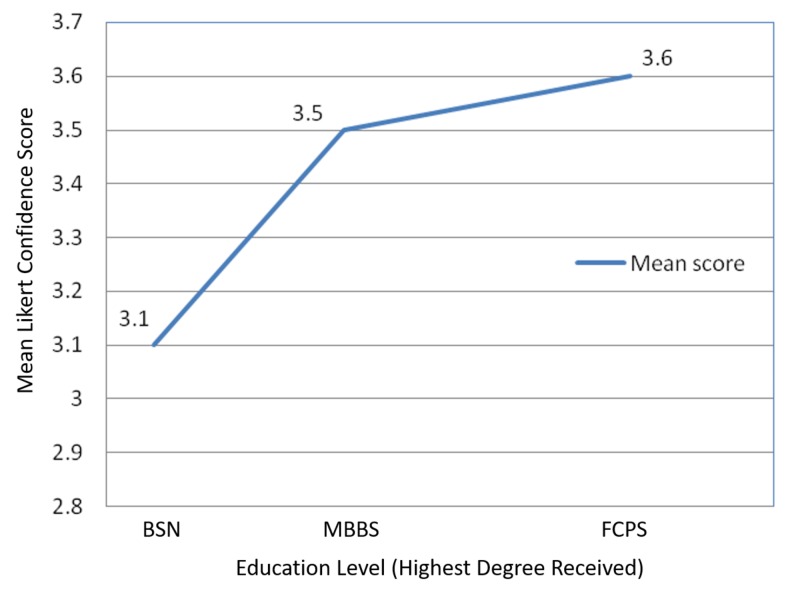
Graph comparing mean Likert scale self-reported confidence scores by education level. BSN: Bachelor’s Degree of Nursing; MBBS: Bachelor of Medicine, Bachelor of Surgery; FCPS: Fellow of College of Physicians and Surgeons Pakistan.

The mean confidence scores of health care workers with MBBS or FCPS qualifications were statistically significantly higher than health care workers with a BSN (Figure 1), when imagining a situation of mass casualties involving an NBC attack/incident. In general, since the 2005 earthquake, respondents with MBBS and FCPS degrees expressed more confidence that their health care institutions were prepared for future earthquakes than respondents with BSNs.

## Discussion

Our findings indicate this institution is not adequately prepared for a mass casualty event such as an NBC incident. These findings were consistent with the previous similar study conducted in Canada [11]. Unfortunately, the goals of terrorism (such as NBC attacks) include spreading feelings of a lack of safety and cultivating a sense of insecurity, chaos, and hopelessness. Therefore, good communication with the public is crucial in this context [12]. A study in Israel reported that special attention should be given to trauma nurses as they suffer more frequently from burnout, intrusive memories, and high levels of stress [13].

In our study, health care workers with MBBS or FCPS reported feeling adequately prepared to deal with a large-scale emergency. Their perceived preparedness scores were higher than those with a BSN; the difference in feelings of preparedness may be due to the differences in experience and education. These findings aligned with those of the Canadian study in which nurses ranked their institutions as having lower scores of preparedness than physicians [11]. However, a review of 18 real-world studies found no significant differences between doctors and nurses in patient outcomes in dealing with an emergency [14].

Moreover, we noted that men rated themselves as more prepared than women when imaging a future NBC incident. As most of the doctors in our study were men, and most of the nurses were women, there may be many underlying causes for these differences related to gender roles and educational expectations in Pakistan. The training practices for women in preparation for a large-scale NBC incident should be further explored to determine the cause for these differences in self-reported preparedness. One study reported that female participants had higher levels of self-reported anxiety and worry regarding an NBC-related mass casualty event [15]. Additional education, preparedness training or improved existing training may mitigate said anxiety and worry. The gender differences evident in our findings may reflect a deeper, multifaceted cultural phenomenon, the exploration of which is outside the scope of this study.

In our study, the majority of surveyed health care professionals (those with BSN, MBBS, and FCPS degrees) reported low levels of perceived institutional adequacy in terms of preparedness for a mass casualty NBC incident. Most health care professionals were not aware of global disease surveillance or the presence of large-scale emergency planning in their institution. These findings were consistent with a study in the United States, in which most nurses were unaware of a mass-scale emergency management plan in their hospitals [16].

To date, Pakistan has not been exposed to any major NBC attack, but the country has faced major terrorist attacks in the past two decades and an earthquake in 2005 that caused 10,000 casualties and 138,000 injuries and rendered 3.5 million people homeless. Devastating floods occurred in 2010 [13]. Pakistan’s demographics and current global political position in times of increased global terrorism (including state-sponsored terrorism) make the threat of an NBC incident imminent. Therefore, high-level changes are critical in large-scale emergency response policies, particularly specific to NBC-incident protocols.

## Conclusions

Health care professionals in Pakistan play a key role in emergency, mass casualty, and disaster management. Physicians reported a higher degree of self-confidence than nurses in handling an NBC incident. Additional training and education for nurses may increase their level of self-reported confidence for perceived NBC incidents. Studies on perceived institutional and professional preparedness are quite useful for future disaster management training. Mass-scale emergency drills and protocols should be part of undergraduate curricula in our medical colleges and nursing programs so that our health care professionals maintain a high degree of self-confidence in extreme circumstances. Our study was limited in that it was conducted in a single tertiary care military hospital, as its cross-sectional nature limits inferences related to temporality and causality. Therefore, our results cannot be generalized to the other health care professional populations. Further research is warranted in this regard.
